# Early Intervention via Stimulation of the Medial Septal Nucleus Improves Cognition and Alters Markers of Epileptogenesis in Pilocarpine-Induced Epilepsy

**DOI:** 10.3389/fneur.2021.708957

**Published:** 2021-09-07

**Authors:** Ali Izadi, Amber Schedlbauer, Katelynn Ondek, Gregory Disse, Arne D. Ekstrom, Stephen L. Cowen, Kiarash Shahlaie, Gene G. Gurkoff

**Affiliations:** ^1^Department of Neurological Surgery, University of California, Davis, Sacramento, CA, United States; ^2^Center for Neuroscience, University of California, Davis, Davis, CA, United States; ^3^Department of Psychology, University of Arizona, Tucson, AZ, United States; ^4^McKnight Brain Institute, University of Arizona, Tucson, AZ, United States

**Keywords:** deep brain stimulation, temporal lobe epilepsy, theta oscillations, cognition, pilocarpine

## Abstract

Over one-third of patients with temporal lobe epilepsy are refractory to medication. In addition, anti-epileptic drugs often exacerbate cognitive comorbidities. Neuromodulation is an FDA treatment for refractory epilepsy, but patients often wait >20 years for a surgical referral for resection or neuromodulation. Using a rodent model, we test the hypothesis that 2 weeks of theta stimulation of the medial septum acutely following exposure to pilocarpine will alter the course of epileptogenesis resulting in persistent behavioral improvements. Electrodes were implanted in the medial septum, dorsal and ventral hippocampus, and the pre-frontal cortex of pilocarpine-treated rats. Rats received 30 min/day of 7.7 Hz or theta burst frequency on days 4–16 post-pilocarpine, prior to the development of spontaneous seizures. Seizure threshold, spikes, and oscillatory activity, as well as spatial and object-based learning, were assessed in the weeks following stimulation. Non-stimulated pilocarpine animals exhibited significantly decreased seizure threshold, increased spikes, and cognitive impairments as compared to vehicle controls. Furthermore, decreased ventral hippocampal power (6–10 Hz) correlated with both the development of spikes and impaired cognition. Measures of spikes, seizure threshold, and cognitive performance in both acute 7.7 Hz and theta burst stimulated animals were statistically similar to vehicle controls when tested during the chronic phase of epilepsy, weeks after stimulation was terminated. These data indicate that modulation of the septohippocampal circuit early after pilocarpine treatment alters the progression of epileptic activity, resulting in elevated seizure thresholds, fewer spikes, and improved cognitive outcome. Results from this study support that septal theta stimulation has the potential to serve in combination or as an alternative to high frequency thalamic stimulation in refractory cases and that further research into early intervention is critical.

## Introduction

Of the ~3.4 million adults and children in the U.S. diagnosed with epilepsy (1), more than half also experience comorbidities such as cognitive decline (2). Temporal lobe epilepsy (TLE) is a frequently diagnosed form of focal epilepsy and also difficult to treat pharmacologically, as antiepileptic drugs fail to control seizures in nearly 40% of cases (3–5). Even when successful, anti-seizure medications are not designed to treat cognitive comorbidities, and in many patients, they induce adverse side effects (6). Surgical resection of the seizure focus can be curative in ~70% of patients (7), however, it is often not performed for 20–25 years after refractory classification, reducing the probability of post-operative seizure freedom and increasing the risk of lasting seizure-related comorbidities, temporal lobe pathology, and injury/fatality (2, 8–11). For patients who do not meet surgical criteria (12), neuromodulation paradigms such as vagal nerve stimulation (VNS), deep brain stimulation (DBS) of the anterior nucleus of the thalamus (ANT), and responsive neurostimulation (RNS) represent reversible and effective alternatives (13–15). While these stimulation paradigms result in progressive improvements in responder rates (13–16) as compared to pharmacology alone, they do not significantly improve cognitive outcome (17).

Animal models of neural connectivity and TLE, predominantly in rodents, have been critical for the development and optimization of electrical neuromodulation. For example, both RNS (18) and DBS of the thalamus (19) were initially tested in animal models including pilocarpine-treated rats prior to clinical translation. In addition, direct stimulation of the hippocampus (20) and the centromedian thalamic nuclei (21) also reduced seizures in the absence of additional cognitive impairments. Critically, the majority of these studies focused on high frequency stimulation parameters to desynchronize neuronal output and reduce epileptiform activity. There are also a limited number of studies demonstrating that low frequency stimulation of the hippocampus (22) and amygdala (23) can reduce seizure frequency. While each of these pre-clinical and clinical findings demonstrate that feasibility of neuromodulation to reduce spontaneous seizures, there remains a critical need for the development of a novel therapeutic paradigm to treat not only seizures but also the significant cognitive impairments associated with TLE.

Low frequency theta oscillations are implicated in both cognitive processing (24–26) and the modulation of seizures (27, 28). We previously demonstrated that 7.7 Hz theta frequency stimulation of the medial septal nucleus (MSN) both increases seizure threshold and improves cognitive function in chronically epileptic rats (29). We now hypothesize that engaging the septohippocampal circuit earlier, in the weeks immediately following pilocarpine injection, will alter the development of epilepsy markers, as measured by spikes and seizure threshold, while concurrently improving long-term cognitive outcome. If early theta stimulation of the septum demonstrates lasting effects, this study would indicate a need to further investigate the potential of closing the gap between diagnosis of refractory epilepsy (~2–3 years) and the consideration for reducing the time from diagnosis to surgical/electrical intervention which frequently takes over 20 years. Moreover, if early stimulation also improves learning, this would represent a single modulatory paradigm that could potentially address both epileptiform and cognitive phenotypes in TLE.

## Materials and Methods

### Experimental Design

**Figure 1:**
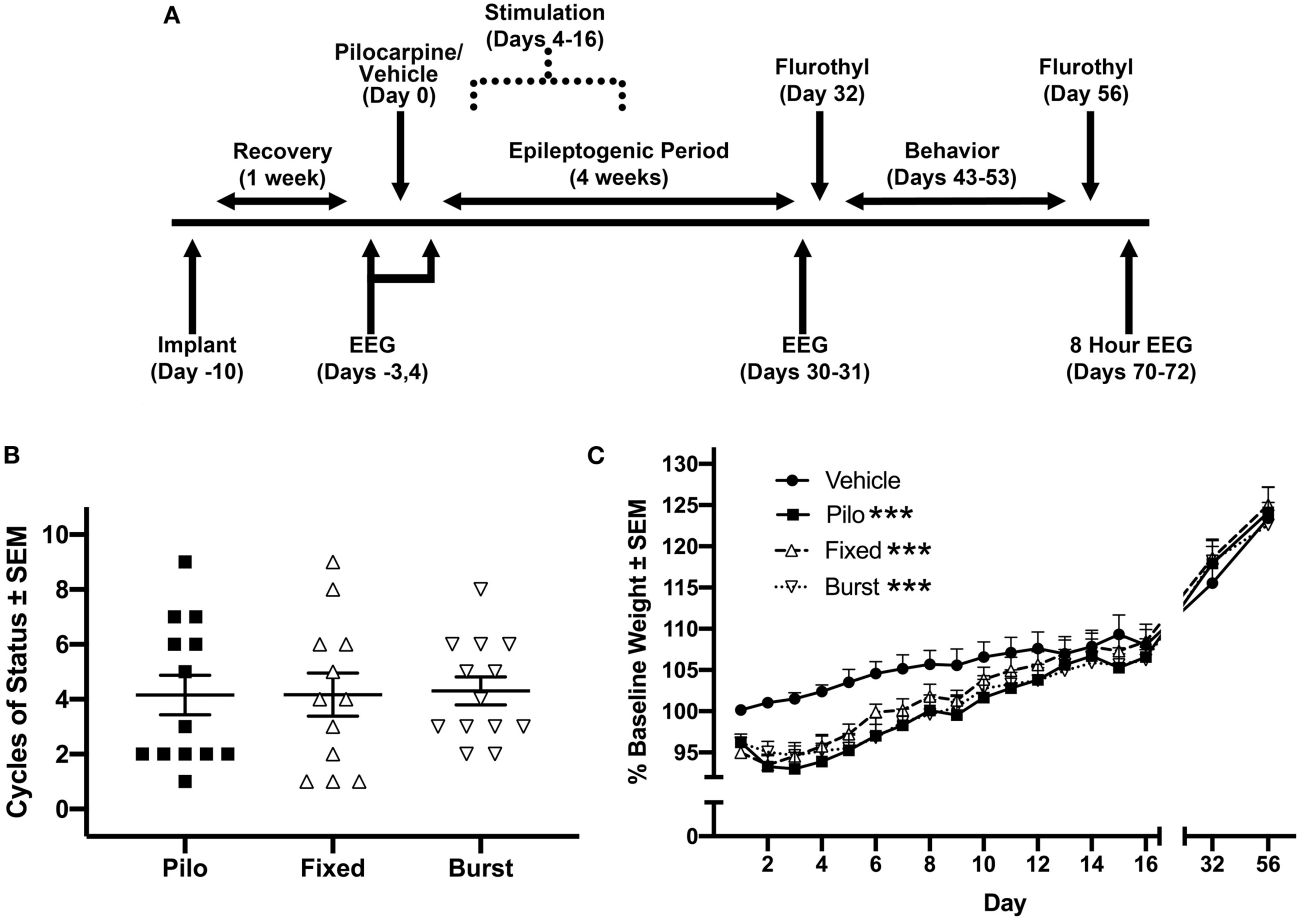
Experimental timeline and biological measures post-pilocarpine injection. **(A)** Timeline of procedures. We allowed 4 weeks, operationally defined as the epileptogenic period, from the time of pilocarpine for rats to develop spontaneous seizures. **(B)** Stimulation groups were counterbalanced via cycles of status epilepticus and **(C)** weight following pilocarpine injection. Each of the three pilocarpine-treated groups were statistically different from vehicle across the first 16 days following pilocarpine administration. However, there were no statistical differences between any of the pilocarpine-treated groups. ***p < 0.001.

All experiments involving animals complied with the ARRIVE guidelines and were performed in accordance with the National Institutes of Health guide for the care and use of laboratory animals and with an approval from the University of California, Davis Institutional Animal Care and Use Committee. Adult male Sprague-Dawley rats (*n* = 75; 300–375 g; Envigo, Livermore, CA, USA) were randomly assigned to vehicle control (*n* = 16) or pilocarpine (*n* = 59); all animals were housed in a standard institutional vivarium with a 12-h (7) light cycle. Complications related to status epilepticus (*n* = 20), and failed implants (vehicle *n* = 2, pilocarpine *n* = 1) resulted in a final sample size *n* = 52. For all behavioral experiments, investigators were blind to group (pilocarpine vs. vehicle) but not to stimulation condition (stimulation is triggered). Therefore, all data was recoded after collection and all analyses were performed blind to condition. A timeline of experiments is illustrated in Figure 1A.

### Electrode Implantation Pre-pilocarpine

Surgical procedures followed previously published work (29). Briefly, animals were anesthetized using 2–4% isoflurane in O2/N2O (1:2) carrier gas, intubated, and mechanically ventilated to maintain a surgical plane of anesthesia. Small craniotomies (2 mm) were made bilaterally over the medial pre-limbic cortex (PFC; AP = +3.2, ML = ±1.3), dorsal hippocampus (dHPC; AP = −3.3, ML = ±2.0), ventral hippocampus (vHPC; AP = −5.2, ML = ±5.3), and MSN (AP = +0.48, ML = ±1.5). Five small stainless steel screws (#0–80) were then embedded into the skull proximal to the craniotomies. Tungsten electrodes (E363T-2-SPC, 0.2 mm diameter; Plastics1, Roanoke VA) were stereotactically implanted in each location including bilateral PFC (DV = −3.5; 9° angle), dHPC and vHPC spanning CA3-CA1 (DV = −3.2, −6.0, respectively), and unilaterally over left MSN (DV = −6.8; 12.8° angle). A pair of electrodes (~1 mm between tips) was also implanted in the right MSN (DV = −7.01; 10° angle) for bipolar stimulation. Electrodes were anchored to the screws using Metabond (Parkell, Inc., Edgewood NY) and non-toxic superglue. Electrodes and screws were subsequently wired (stainless steel) and pinned to separate channels on a Neuralynx electrode interface board (Neuralynx EIB-QC-16). The implant was anchored to the skull with methyl methacrylate and cyanoacrylate adhesive (Yates Motloid, Chicago IL) to form a rigid implant. All animals, including vehicle and pilocarpine-treated rats, underwent identical electrode implantation. Animals recovered for 10–14 days prior to injection of pilocarpine.

### Pilocarpine Administration

As detailed in previous work (29), rats received injections (i.p.) of 1 mg/kg scopolamine methyl nitrate followed 30 min later by 350 mg/kg pilocarpine hydrochloride. Four hours later, animals were injected with 8 mg/kg diazepam to terminate convulsions. Vehicle control animals received similar doses of scopolamine and diazepam, however pilocarpine injection was replaced with an equal dose of sterile saline solution. All pilocarpine-induced rats experienced at least one cycle of status epilepticus as defined by generalized motor convulsions including rearing and falling with forelimb clonus. Animal weights were recorded on days 1–16 following pilocarpine, and again on days 32 and 56.

### Stimulation (PPD-4–16)

Rats were counterbalanced into treatment groups based on a combination of average weight from post-pilocarpine day 1–3 (PPD1–3; Figure 1B) and the number of cycles of status epilepticus in the first 2 h after pilocarpine injection (Figure 1C). Control animals included vehicle controls for pilocarpine with no stimulation (Vehicle; *n* = 14) and pilocarpine-treated rats with no stimulation (Pilo; *n* = 13) rats. Experimental pilocarpine groups received either 7.7 Hz theta (Fixed; *n* = 12) or theta-burst stimulation (Burst; *n* = 13). On PPD4–16, animals were placed in a Plexiglas box (28 × 28 × 30 cm), and bipolar MSN stimulation was applied (IZ2 Stimulator; Tucker Davis Technologies) for 30 min/day. Based on previous studies, stimulation parameters were as follows: (Fixed) 7.7 Hz at 80 μA with 1 ms pulse-width (24, 29–33); (Burst) 50 ms trains of 200 Hz, 5 trains/s, 60 μA, 100 μs pulse-width (34).

### Spike and Seizure Detection, Local Field Potentials (LFPs), and Spectral Analyses

All LFPs were recorded with a head stage pre-amplifier (Neuralynx HS-16-QC) tethered to a data acquisition system (Neuralynx Digital Lynx SX; band-pass filtered 1–500 Hz). Custom MATLAB (MathWorks) scripts were used for signal processing and spike detection. LFP recording sessions were visually inspected, and artifacts were manually rejected prior to analyses. Raw data were then down-sampled to 1,000 Hz and filtered for 60 Hz line noise using a Butterworth bandstop IIR filter.

A custom Matlab script was developed to quantify spikes in PFC, vHPC, and dHPC, as well as the MSN, during novel object recognition sessions. A two-step process was employed to identify epileptiform spikes within each channel for each recording session and each animal. First, amplitude criteria were used to distinguish the positive and negative polarity peaks using the Matlab function *findpeaks()*; the minimum peak height for selection as a spike was defined as 4.5 standard deviations from the signal mean calculated over the entire 5 min recording session. However, when variance was low within an epoch, most often in vehicle controls, and a resulting value was <0.5 mV, the minimum peak height was then reset to 0.5 to reduce physiologic oscillatory activity being mischaracterized as a spike. To limit the number of false positives, a second criterion using increases in power of the signal, was employed to refine the detection approach. Using spectral decomposition, the power was calculated for each channel and epoch. We then averaged the power (normalized within frequency band) across frequencies to obtain a single value for each time point. Again, the peaks in power were detected using a threshold minimum of the 90th quantile of the average power time-series (In this case, standard deviation was not used due to a non-parametric distribution of power values). Only spikes that met both the amplitude and power criteria were considered as spikes. Overall, while there was slight variation in detection performance by the developed algorithm (i.e., inclusion of some false positives and negatives), using a fully automated method ensured no bias in this quantification. Finally, the rates of spikes (spikes/min) were calculated; if there were multiple recording sessions in a day (e.g., the NOR task), the spike rate was averaged over sessions to obtain a single value per day per animal. Following detection and quantification, spikes were rejected prior to spectral analyses.

We also conducted 24 h (3 × 8 h) of seizure monitoring on PPD70-72 in a subset of animals (*n* = 17), as equipment necessary to record simultaneously from multiple rats was acquired midway through the experiment. Seizures were manually identified via video and electrophysiology.

LFP recordings occurred throughout the experimental timeline (Figure 1A), including for 5 min on non-behavioral days in a black Plexiglas box (28 × 28 × 30 cm), and for 5 min prior to and 10 min following stimulation sessions (Baseline, PPD4–16), and during the novel object task and Barnes maze.

Analysis of theta power (6–10 Hz) was performed across multiple recording sessions (pre-pilocarpine, PPD4, PPD31). Spectral power estimates across frequency bands (2–30 Hz, 30 logarithmically spaced points) were calculated using Morlet wavelets (cycles = 6) (35). Both baseline (pre-pilocarpine) and PPD4 power spectral densities were normalized to decibels using the ratio of power in a 15 s epoch of ambulation to the entire 5-min baseline recording session (36). For PPD31, the entire PPD4 recording was used for normalization.

### Flurothyl Testing (PPD32 and 56)

All animals including Vehicle control rats underwent flurothyl testing (29). Seizure threshold was measured using the volatile GABA_A_ antagonist flurothyl (bis(2,2,2-trifluroethyl) ether, Sigma Aldrich) (37). An initial seizure threshold was determined prior to behavioral assessment on PPD32, and a final measurement was obtained on PPD56, after cognitive evaluation. Flurothyl was dripped directly onto filter paper at a rate of 1.2 mL/h (29). Times to induce Racine stages 1–5 seizures were recorded and used to compare individual thresholds. PPD32 and 56 values were compared to determine effects of time and stimulation.

### Novel Object Recognition (NOR) Task (PPD43–45)

On PPD43, animals were habituated for 5 min to a dark, square Plexiglas box (100 × 100 × 40 cm) with cues on each wall. On PPD45, animals were placed in the box for 5 min with two of three potential objects positioned 40 cm apart. Animals were then placed in a neutral cage for 3 h and subsequently returned to the box for a 5 min test phase, in which one of the objects was replaced with a novel object. Objects and object positions were randomized. Recognition index, a comparison of the percentage of time animals interacted with the novel object relative to overall object interaction, was calculated (38).

### Barnes Maze (PPD49–53)

The Barnes maze consists of a Plexiglas table (145 cm diameter) with 22 peripheral holes, a goal box in a fixed location under one hole, and four equally spaced distal cues around a dark curtain. Barnes maze habituation occurred on PPD49, and testing spanned PPD50–53 (2 trials/day; 8 total trials). Escape latency and search strategy (spatial, peripheral, or random) were evaluated on trials 2–8. Spatial strategy was defined as the use of a direct path to the goal box; peripheral strategy was defined as circling the periphery of the table; and random search strategy was defined as exploration of non-consecutive holes. Animals tended to forage and explore the Barnes maze on the first trial due to its novelty. As a result, the first trial was omitted from the analysis. Parameters and analyses were based on previous studies (29, 32, 33).

### Statistical Analyses

Except where noted, all statistical data are presented as the mean ± SEM. Statistical significance was assigned to values *p* < 0.05. All statistical analyses were conducted in GraphPad Prism 8.1.

PPD1–16 weight changes across groups were evaluated using a mixed-model ANOVA with a Bonferroni *post-hoc* analysis. A one-way ANOVA was used to evaluate group differences in cycles of status following pilocarpine.

A one-way ANOVA was used to compare spike counts with a *post-hoc* Dunnett's multiple comparisons test comparing each of the pilocarpine-treated rats to the Vehicle control. To evaluate the effect of stimulation on seizure threshold, a mixed-effects ANOVA was performed, comparing groups across the two time points (PPD32, 56); a *post-hoc* Dunnett's multiple comparisons test was utilized to compare all groups to Vehicle.

For analysis of oscillatory activity, including pre-pilocarpine, PPD4, and PPD31, separate mixed model ANOVAs were utilized to compare the theta power spectral data between groups across delta (2–5 Hz), theta (6–10 Hz), and beta (12–30 Hz) frequencies. A *post-hoc* Dunnett's multiple comparisons comparing all groups to Vehicle was used to determine whether there was a difference between counter-balanced groups prior to stimulation on PPD4 and also whether there was a change in oscillatory power between PPD4 and PPD31. A subset of animals was not included in electrophysiological analyses due to noise resulting from pilocarpine induction as well as channel dropout over time.

To evaluate NOR, a one-way ANOVA with a Dunnett's multiple comparisons test was used to compare all treatment groups to Vehicle. Several animals were excluded due to seizures either immediately before or during the task (*n* = 4) or for failure to explore the objects during acquisition (*n* = 5).

Average daily Barnes maze latency was calculated, and a repeated-measures ANOVA was used with a Dunnett's *post-hoc* test to compare all treatment groups to Vehicle. A non-parametric chi-square analysis was used to compare differences in search strategies (spatial, peripheral, random) across the sum of all trials, with Vehicle performance used as the test proportion. All analyses excluded trial 1, as animal performance was necessarily random/unlearned. Four animals were not included due to seizures.

Separate linear correlation analyses were performed to determine the relationship between: PPD31 power × total Barnes maze latency (trials 2–8); PPD31 power × spikes during NOR. As described above, several trials were removed from the analysis due to seizures as well as channel noise/dropout over time.

### Histology

Upon completion of the experiment, animals were euthanized (Euthasol, Virbac Corp., Fort Worth, TX) and transcardially perfused with 4 C 0.1M sodium phosphate buffer (pH 7.4; 100 mL) followed immediately by 4 C 4% paraformaldehyde (pH 7.4; 350 mL). Brains were removed and post-fixed for 24 h in 4% paraformaldehyde at 41 C, then immersed in 10% sucrose solution for 24 h, followed by 48 h in a 30% sucrose solution. After buffer exchange, electrode tract locations were confirmed for all animals in the study using previously described histological methods (29, 32, 33) to visualize NeuN (MAB377 MilliporeSigma, Burlington MA).

## Results

### Pilocarpine-Induced Seizures and Epileptogenesis

All animals injected with pilocarpine experienced motor limbic seizures followed by status epilepticus. Counterbalanced pilocarpine-treated groups did not significantly differ from each other in terms of cycles of status epilepticus following exposure to pilocarpine (Figure 1B). There was a significant effect of group (*F*_(3, 48)_ = 2.88, *p* = 0.046) and an interaction between group and time (*F*_(45, 680)_ = 2.15, *p* < 0.0001) on PPD1–16 weight (Figure 1C). *Post-hoc* Bonferroni analysis demonstrated that each pilocarpine group was statistically different from Vehicle (*p* < 0.0001 for all comparisons), however there were no differences between Pilo, Fixed, or Burst groups. By days 32 and 56, there were no differences in weight between any of the groups.

### Spikes Are Evident Across Groups

**Figure 2:**
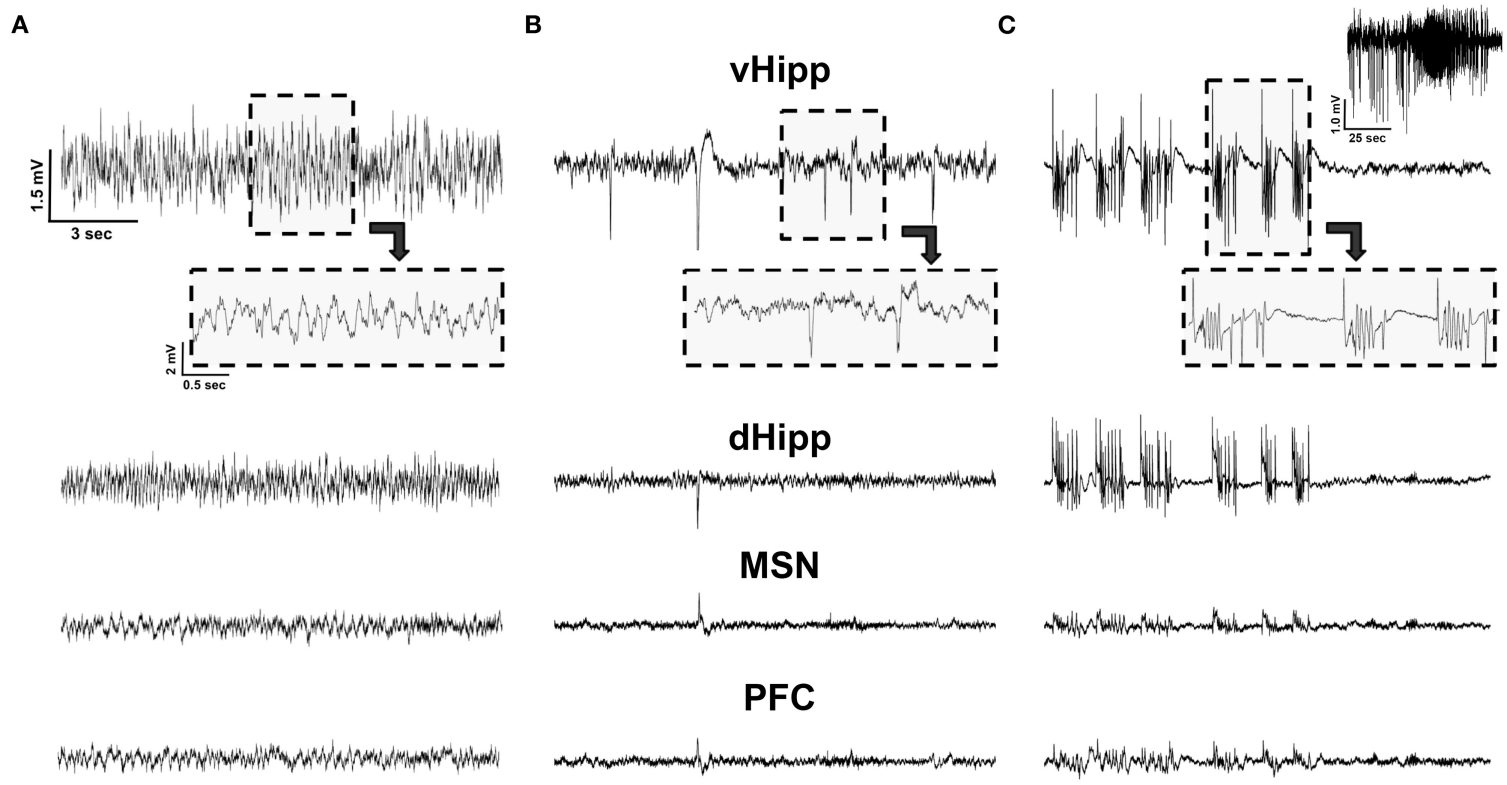
LFP recordings. **(A)** An example of LFP traces in a Vehicle animal from the ventral (vHPC) and dorsal (dHPC) hippocampus, medial septal nucleus (MSN), and pre-frontal cortex (PFC). **(B)** Pilocarpine-injected animals exhibited spikes; all detected spikes could be observed in the vHPC, with only a subset detected in the more remote MSN and PFC. **(C)** Pilocarpine-injected animals also exhibited spontaneous seizures. This electrographic activity represents a visually confirmed seizure with the entire seizure recorded from the vHPC depicted on the top right. Note, while the activity was highly synchronized and observable across channels, seizure amplitude was maximal in the vHPC and was minimal in MSN and PFC. All traces are on the same scale.

**Figure 3:**
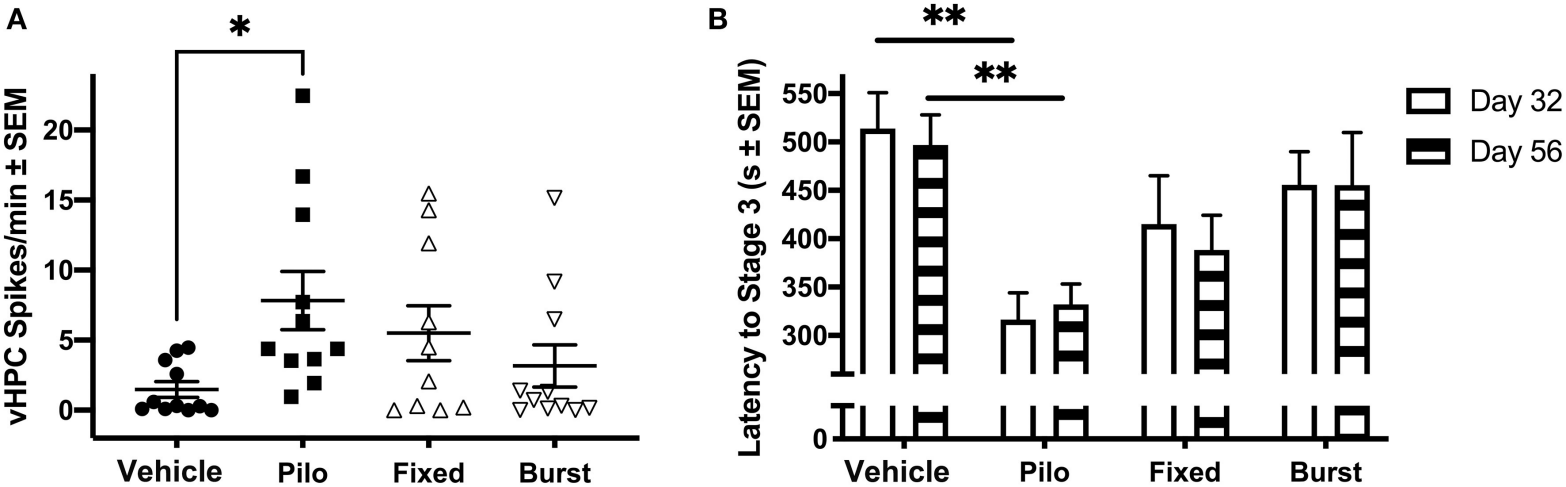
Ictal activity in epileptic animals. **(A)** During the novel object recognition task, there was a significant difference in spike rate when comparing Pilo animals with Vehicle. **(B)** On PPD32 and 56, Pilo animals demonstrated significantly reduced stage 3 seizure threshold as compared to Vehicle animals. Both stimulation groups had similar seizure thresholds to the Vehicle group. *p < 0.05, **p < 0.01.

We hypothesized that stimulation would reduce spikes. While regular oscillations were observed across the vHPC, dHPC, PFC, and MSN in Vehicle animals (Figure 2A), there was evidence of spikes (Figure 2B) and seizures (Figure 2C) across regions in pilocarpine-treated rats. While all regions were analyzed (Table 1), data presented represent observations in the vHPC as changes in number and amplitude of spikes were greatest in this region. There was a significant main effect of group in vHPC spike rate (*F*_(3, 39)_ = 2.957, *p* = 0.044). Dunnett *post-hoc* analysis indicated that Pilo animals had significantly higher spike rates than Vehicle (7.82 ± 2.07 vs. 1.48 ± 0.56 spikes/min, *p* = 0.022). Critically, following either Fixed or Burst stimulation (5.50 ± 1.96, *p* = 0.22 and 3.16 ± 1.51, *p* = 0.80, respectively), spike rates remained statistically similar to Vehicle (Figure 3A; Table 1). Over the 24 h (3 × 8 h) of seizure monitoring of a small subset of the animals (data not shown), there were no differences in the number of seizures between pilocarpine groups.

**Table 1:** **∫**Average spike rate (spikes/min ± SEM) during the novel object task.

	**Vehicle**	**Pilo**	**Pilo-FT**	**Pilo-TB**
dHPC	1.23 ± 0.34	3.3 ± 1.08	1.81 ± 0.67	3.57 ± 1.27
PFC	0.48 ± 0.19	1.17 ± 0.33	0.73 ± 0.25	3.08 ± 1.11
MSN	0.71 ± 0.3	1.1 ± 0.42	3.34 ± 1.9	0.5 ± 0.18

### Early Stimulation Has Lasting Effects on Seizure Threshold

To determine if there were persistent effects of stimulation on seizure susceptibility, seizure threshold was evaluated on PPD32 and 56 (Figure 3B). As seizure threshold values were co-linear between groups, and statistical differences were similar across seizure stages, Racine stage 3 was selected as an example. A mixed-effects ANOVA comparing latency to Racine stage 3 indicted a significant main effect of group [*F*_(3, 47)_ = 5.77, *p* = 0.002]. A Dunnett's test demonstrated that Pilo animals had a significantly reduced stage 3 threshold compared to Vehicle on both PPD32 (316.46 ± 27.74 vs. 513.92 ± 37.05 s, *p* = 0.002) and PPD56 (332.23 ± 20.94 vs. 496.85 ± 31.19 s, *p* = 0.014). Similar to what was observed when quantifying spikes, rats receiving Fixed (415.08 ± 49.96 s, *p* = 0.35 on PPD32; 388.33 ± 35.92, *p* = 0.25 on PPD56) or Burst (455.62 ± 34.43, *p* = 0.85 on PPD32; 455.46 ± 54.46 s on PPD56, *p* = 0.97) stimulation were not statistically different from Vehicle. Analyses of seizures stages (stages 1, 3, and 5) are presented in Table 2.

**Table 2:** Seizure thresholds across the Racine scale.

		**Day 32**			**Day 56**		
**Group**	**(** ***n*** **)**	**Stage 1**	**Stage 3**	**Stage 5**	**Stage 1**	**Stage 3**	**Stage 5**
Vehicle	14	478.2 ± 34.2	513.9 ± 37.0	529.2 ± 38.4	480.9 ± 33.2	496.8 ± 31.2	508.2 ± 29.5
Pilo	13	277.6 ± 30.2	316.5 ± 27.7	347.9 ± 33.5	290.0 ± 24.6	332.2 ± 20.9	403.7 ± 33.9
Fixed	12	368.2 ± 35.3	415.1 ± 50.0	447.7 ± 61.0	338.8 ± 33.2	388.3 ± 35.9	446.4 ± 55.7
Burst	13	385.1 ± 26.6	455.6 ± 34.4	481.5 ± 37.1	379.2 ± 29.0	455.5 ± 54.5	510.8 ± 55.0

### Status Epilepticus Suppresses Broadband Activity

**Figure 4:**
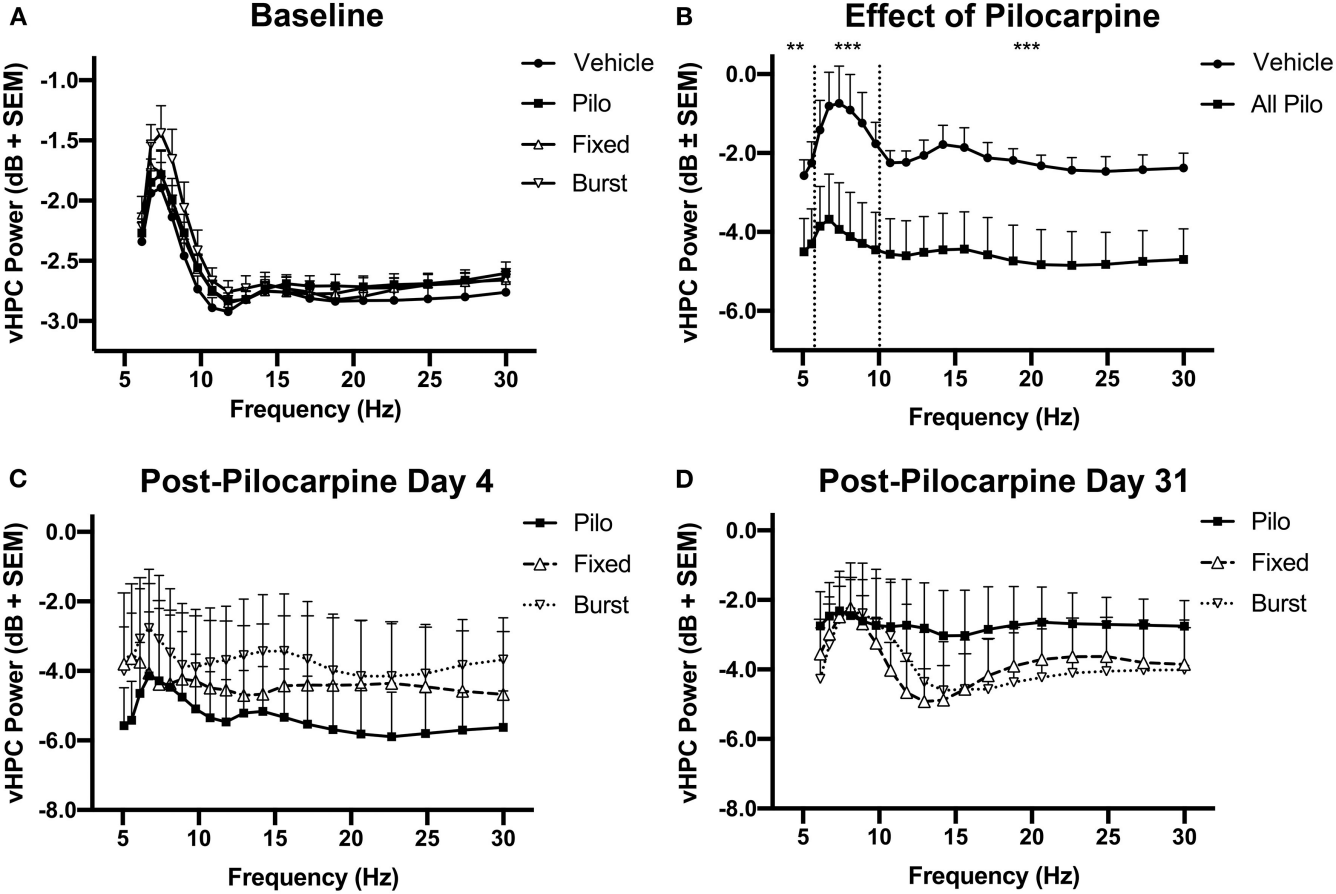
Longitudinal changes in vHPC power. **(A)** Prior to the administration of pilocarpine (baseline), oscillatory power was similar between the groups. **(B)** However, theta, delta, and beta power significantly decreased on PPD4 (normalized to baseline) in animals treated with pilocarpine as compared with Vehicle. **(C)** Animals counterbalanced into stimulation groups demonstrate no difference in 6–10 Hz power prior to the initiation of stimulation (PPD4). **(D)** When normalized to PPD4, PPD31 power was not significantly different between treatment groups. **p < 0.01, ***p < 0.001.

Prior to pilocarpine injection, there were no differences in vHPC oscillatory power between all animals based on eventual grouping (Figure 4A). We hypothesized that reduced theta oscillations would be evident following pilocarpine injection. Four days following pilocarpine but prior to stimulation, there was a significant decrease in vHPC theta power as compared to Vehicle (Figure 4B; *F*_(1, 192)_ = 14.52, *p* = 0.0002). There was also significant suppression of the neighboring delta (*F*_(1, 384)_ = 9.749, *p* = 0.002) and beta (*F*_(1, 320)_ = 27.34, *p* < 0.0001) bands, indicating that the response to pilocarpine over the first days following status epilepticus suppressed broadband spectral activity.

Pilocarpine animals were counterbalanced into Pilo, Fixed, and Burst and there was no difference in vHPC power between the three pilocarpine-treated groups on PPD4 (Figure 4C) prior to stimulation. Comparing power of PPD31 to PPD4, it was clear that theta oscillations remained depressed in animals treated with pilocarpine, and that there were no lasting benefits of stimulation on oscillatory power (Figure 4D).

In the dHPC, significant differences in the theta [*F*_(1, 256)_ = 13.36, *p* = 0.0003] and beta [*F*_(1, 352)_ = 26.22, *p* < 0.0001] bands, but not delta [*F*_(1, 384)_ = 3.336, *p* = 0.069], were apparent between groups on PPD4; similar to the vHPC, there were no differences on PPD31 (data not shown). For PFC, significant group differences were only apparent in the beta [*F*_(1, 280)_ = 65.80, *p* < 0.0001] band on PPD4 (data not shown).

### Early Stimulation Improves NOR and Barnes Maze Performance

Animals were tested in the NOR and Barnes Maze to identify any persistent effects of early stimulation on object-based and spatial memory performance during the chronic stage of epilepsy.

#### Novel Object Task

**Figure 5:**
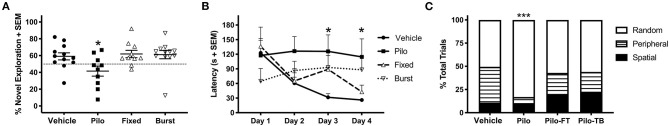
Group differences on behavioral tasks. **(A)** On PPD 43–45, Pilo animals demonstrated significantly reduced preference for the novel object as compared to Vehicle. Fixed and Burst stimulation groups were no different than Vehicle. **(B)** Analysis of the Barnes maze on PPD 50–53 revealed that Pilo animals had a significantly elevated latency to the escape box as compared with Vehicle. Both stimulation groups had statistically similar latencies to the Vehicle group. **(C)** Pilo animals demonstrated significantly different search strategy utilization as compared with Vehicle. Both stimulation groups also had different strategy utilizations as compared to Vehicle. *p < 0.05, ***p < 0.001.

As increased preference for the novel object indicates memory recognition (38), we hypothesized that epilepsy would result in reduced exploration of the novel object. Analysis via one-way ANOVA revealed a significant group difference in performance (Figure 5A, *F*_(3, 40)_ = 3.630, *p* = 0.02); importantly, a Dunnett's *post-hoc* test indicated that Pilo animals had significantly reduced preference for the novel object (41.5% ± 6.0; *p* = 0.041) compared to Vehicle animals (59.0% ± 4.2). Both Fixed (61.9% ± 4.4, *p* = 0.96) and Burst (61.1% ± 4.9, *p* = 0.98) stimulation groups exhibited a clear preference for the novel object similar to Vehicles.

#### Barnes Maze

We identified a significant group-by-time interaction on Barnes maze escape latency (Figure 5B; *F*_(9, 132)_ = 2.316, *p* = 0.019) indicating a difference in how the groups learned across days. While all the animals performed similarly on days 1 and 2, a Dunnett's *post-hoc* analysis demonstrated that Pilo animals had significantly longer latencies to the goal box as compared to Vehicle by days 3 (125.86 ± 33.84 vs. 31.64 ± 7.65 s; *p* = 0.023) and 4 (114.59 ± 36.99 vs. 26.00 ± 3.71 s; *p* = 0.035). Both Fixed and Burst demonstrated an intermediate phenotype and were statistically similar to Vehicles across all days.

Search strategy is an important indicator of learning during spatial navigation. Pilo animals predominantly used random search strategies (83.1%) compared to Vehicle (50.5%), Fixed (57.1%), and Burst (56%) rats. Therefore, similar to Vehicle, Fixed, and Burst animals utilized more efficient peripheral and spatial strategies (Figure 5C). Using Vehicle as the test proportion, non-parametric chi-square analyses demonstrated significantly worse search strategies in Pilo rats [χ^2^(2) = 40.06; *p* < 0.0001].

### vHPC Theta-Band Power Is Correlated With Cognitive Performance and Spikes

**Figure 6:**
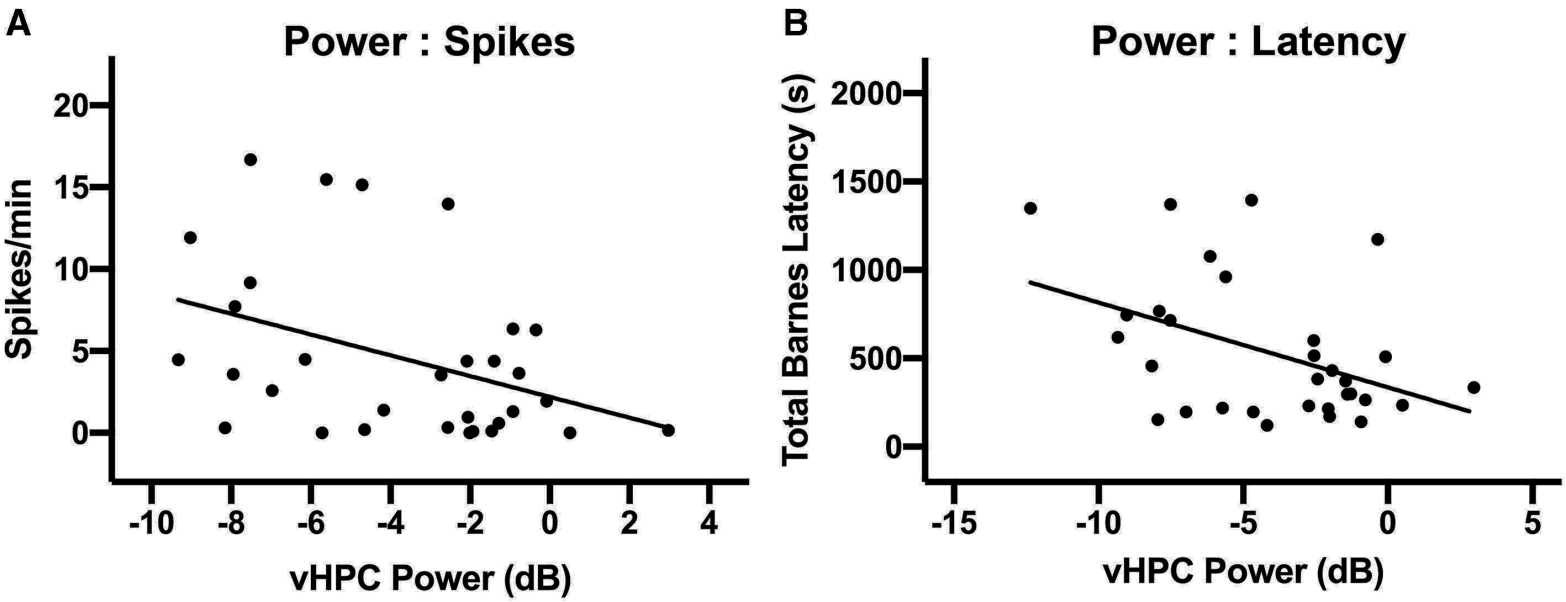
Relationship between vHPC power, behavioral performance, and spikes. **(A)** There was a significant linear relationship between vHPC power (6–10 Hz) on PPD31 and the development of spikes (*p* < 0.05). **(B)** vHPC Power (6–10 Hz) on PPD31 was also significantly correlated with total Barnes maze latency (*p* < 0.05).

We predicted that there would be a strong relationship between theta oscillatory power on PPD31 and both epileptiform activity and Barnes maze performance. A correlation matrix identified a significant relationship between theta power and spikes, with reduced theta power in the vHPC associating with elevated spike counts (Figure 6A, *r* = −0.39, *p* = 0.027). In addition, we found that higher theta power correlated with shorter latencies to the escape box in the Barnes maze (Figure 6B, *r* = −0.43, *p* = 0.015), indicating that theta oscillations in the vHPC and spatial navigation are also directly associated. In the dHPC, a similar relationship between power and spikes (data not shown, *r* = −0.47, *p* < 0.0081), and power and latency (data not shown, *r* = −0.51, *p* = 0.0018) was also observed.

## Discussion

In the current study, we evaluated whether theta stimulation during early epileptogenesis could alter the progression of pilocarpine-induced TLE. Critically, our results indicate that 2 weeks of daily stimulation led to lasting changes. Specifically, while Pilo rats had significantly higher spike rates, lower seizure thresholds, and were impaired on the NOR and the Barnes maze, rats receiving Fixed or Burst stimulation performed statistically similar to Vehicle across all measures in the weeks following stimulation. These novel results further corroborate research from other labs (39), and also add to our previous findings that theta-frequency stimulation of the MSN when administered at the time of behavioral testing improves spatial learning in the first 2 weeks following status epilepticus (32) and also in chronically epileptic rats (29). Ultimately, this novel therapeutic paradigm of acute septal theta stimulation represents a paradigm with the potential to not only alter the development epileptiform activity, but also to improve long-term cognitive outcome.

### Fixed-Frequency and Theta-Burst Stimulation of the MSN

Theta oscillations facilitate the timely integration of activity from multiple brain regions and are important in the brain's ability to form and recall memories (26, 31). In rodents, lesions of the MSN, or septal suppression with pharmacological agents such as tetracaine, scopolamine, or muscimol, all attenuate hippocampal theta oscillations and are associated with significant cognitive deficits (25, 31, 40–42). In epilepsy, we see a similar set of relationships between septohippocampal abnormalities (i.e., reduced theta oscillations), and impaired cognition. Specifically, LFP recordings from epileptic rats reveal a significant reduction in hippocampal theta power and amplitude (27, 29) and demonstrate impaired performance in memory tasks both acutely following induction (32, 43) and after developing SRS (43–45). Our data add to these prior findings as we now report statistically significant correlations between a reduction in theta power and an increase in spikes as well as between decreases in theta power and longer latencies on the Barnes maze. While we have previously indicated the benefits of theta MSN stimulation after animals have developed spontaneous seizures, data from this novel paradigm indicate that stimulated animals (Fixed and Burst) performed at Vehicle-level on both the NOR and the Barnes maze when stimulation was applied early, and behavior was assessed weeks after stimulation was terminated. More specifically, on the NOR stimulated animals had similar preference for the novel object (Vehicle 59.0% ± 4.2, Fixed 61.9% ± 4.4, and Burst 61.1% ± 4.9) and when evaluating spatial memory on the Barnes maze, search strategies utilized by stimulated animals indicated significantly more efficient learning, similar to Vehicle (Vehicle 11% Spatial, 38.5% peripheral, Fixed 19.5% spatial, 23.4% peripheral, and Burst 22.6% spatial, 21.4% peripheral).

In addition to fixed theta stimulation, we also chose to evaluate theta burst stimulation of the MSN. While theta burst stimulation is associated with superior long-term potentiation compared to simple fixed-frequency stimulation paradigms (46), we were most interested in the relationship between burst stimulation and intrinsic septohippocampal activity. It is well-established that the MSN is critical for modulating hippocampal excitation and inhibition (47). More specifically, it is hypothesized that bursting septal neurons pace the oscillatory frequency of theta fields in the hippocampus (48–50). This is evidenced by both the temporal precedence of MSN bursting neurons and their tight phase locking with the ongoing hippocampal theta fields (49–51). In addition, following pilocarpine, changes in hippocampal theta oscillations coincide with altered neural discharges and burst rates in the MSN (27, 52–54) resulting in the phase relationship between septal activity and the hippocampal theta rhythm becoming uncoupled (52, 53, 55, 56). For these reasons, we evaluated both 7.7 Hz fixed-frequency and burst stimulation of the MSN. Interestingly, we did not find any significant differences between paradigms as they pertained to spikes or cognitive outcomes in this study.

### vHPC Theta Power Is Associated With Spikes and Cognitive Performance

We consistently observed the highest amplitude seizures and the most spikes originated in the vHPC. Moreover, we found that oscillations in the vHPC significantly correlated with outcome. Specifically, chronic oscillatory changes in the vHPC weeks following pilocarpine exposure were significantly associated with spikes and impaired spatial learning.

Observed relationships between vHPC theta and increased spikes are in line with both preclinical and clinical observations, demonstrating the importance of this region in epileptogenesis. For example, a recent study evaluating ictogenesis in 600 pilocarpine-induced seizures across 16 bilaterally distributed electrode locations found that, while seizures usually generalized to all regions, they frequently originated in the vHPC (57). Similarly, in one clinical case series, 80% of patients with TLE had a seizure focus either in the anterior portion of the hippocampus (vHPC in the rat) or amygdala (58, 59). Furthermore, detection of pathological oscillations in temporal regions such as the anterior hippocampus during intraoperative electrocorticography has become a valuable predictor of successful outcome following resection of potentially epileptogenic tissue (60).

The role of vHPC in cognition is also well-described. The vHPC and PFC are monosynaptically connected, and functional communication between these structures is critical for working memory in the rodent (61–64). Furthermore, individual neurons in the PFC phase-lock with ongoing theta field activity in the hippocampus via the ventral subregion during spatial, non-spatial, and object-based behavioral tasks (62–64). Importantly, disruption of neural activity in the vHPC, whether surgically or resulting from pilocarpine induction, results in impaired working memory and reduced theta oscillations (29, 32, 43, 44, 65). As such, it is not surprising that, in the current study, animals with higher power theta oscillations were consistently better performers on the Barnes maze.

While it is evident that reduced theta power is correlated with increased spikes and impaired cognition, it remains unclear the mechanism with which stimulation results in better cognitive performance. While we were not able to quantify the effects of stimulation on seizure frequency, both Fixed and Burst-treated rats exhibited similar seizure thresholds and rates of spikes compared to Vehicle controls. Because spikes are believed to interfere with learning and memory, fewer spikes or increased seizure threshold as a result of stimulation may in turn have contributed to improved cognition regardless of a change in seizure frequency (66). Conversely, stimulated animals may have also exhibited better performance directly as a result of stimulating critical cognitive circuits functionally connected with the MSN (24, 25, 67–69) leading to lasting plasticity. Low frequency stimulation of the MSN may be in fact driving oscillatory activity in both proximal and more distally connected regions such as the hippocampus and PFC, where oscillations may be disturbed as a result of epileptogenesis. Studies from both our lab and others have shown that low frequency stimulation of the MSN, either continuously or immediately before behavioral testing, results in improved outcome in various injury models including pilocarpine, traumatic brain injury, as well as in lesion studies (29, 31–33). Interestingly, a recent study of low frequency 1 Hz hippocampal stimulation identified similar cognitive benefits in pilocarpine-treated rats (70). Ultimately, these data highlight the importance of further investigation into the mechanisms of how DBS impacts hyperexcitability and cognition, particularly over extended periods of time.

### Argument for Earlier Intervention

TLE is considered refractory after two failed drug treatments (10, 71). Although resection has a high rate of seizure freedom (~70%), surgical options remain a last resort, often occurring more than 20 years after disease onset (7, 72–74) and continued failure to respond to medicine (75). TLE, however, can be progressive, resulting in debilitating consequences such as hippocampal sclerosis and death, especially if seizures are poorly controlled (72, 76, 77).

When resection is not an option (12), FDA-approved neuromodulation paradigms, including DBS of the ANT, RNS, and VNS, all result in ~70% seizure reduction over time. In addition, neuromodulation has fewer irreversible consequences than resection (13–15). Current epilepsy stimulation paradigms, however, also occur as a “last resort,” decades after diagnosis, and do not effectively resolve TLE-associated cognitive deficits. Several clinical examples in childhood and adolescent epilepsies emphasize the potential for earlier intervention. Younger patients receive surgical intervention more rapidly, due to the greater impact of seizures on development and also based on evidence of high rates of seizure freedom resulting from surgery (78, 79). In a retrospective study comparing surgical outcome in children and adults with intractable TLE, a combined 80% of children, compared to 49% of adults, were seizure free despite similar rates of hippocampal sclerosis (80). Furthermore, the likelihood of seizure freedom following surgery is significantly increased in patients with fewer than 5 years of epilepsy history (80), indicating that not just age but also intervention more proximal to diagnosis increased treatment efficacy. Critically, the results of our study support that (1) Early intervention in rodents can have a lasting effect including a reduction in spikes and an increase in seizure threshold; (2) DBS of the MSN has the potential to concurrently improve cognitive outcome. In sum, data from our lab as well as clinical studies support the potential benefits of early intervention. While current anti-seizure treatment strategies should necessarily continue as the first line of treatment, we argue for additional studies to explore the more expeditious implementation of DBS in refractory patients (i.e., to close the gap between diagnosis and surgical referral) as well to strategies to address epilepsy comorbidities.

### Study Limitations and Future Directions

While we were able to utilize seizure threshold and spikes as proxy measures of increased hyperexcitability and epileptogenesis, due to technological constraints, we were unable to continuously record from our animals. With the recordings we did make, we lacked sufficient power to make a claim about the effects of stimulation on the development of seizures. As pilocarpine-induced epilepsy typically results in an average of 2–3 spontaneous seizures per week (81, 82), 24 h of total recording over a 3 day period, during the light cycle only, and in only a subset of animals resulted in a significant under-sampling. Therefore, we do not report any conclusive results regarding the effect of stimulation on seizure occurrence. However, our pilocarpine induction procedures have been well-characterized in previous studies and all of our animals experienced status epilepticus following pilocarpine (29, 32, 81, 83–87). It is critical, therefore, that future studies incorporate continuous monitoring to determine whether stimulation-induced changes in spikes and seizure threshold result in a significant reduction in seizure frequency.

We developed a custom Matlab script to identify spikes objectively and in an unbiased manner. This included counting any oscillatory activity that either (1) exceeded a 4.5 standard deviation change from baseline, or, in the case where there was little variance, such as in vehicle controls, (2) exceeding a minimum peak height. The result was that, in several of our vehicle controls, some spiking activity above baseline was detected. Rather than manipulate the code to eliminate spikes in our controls, or not show the vehicle data all together, we elected to keep the unbiased analysis in the manuscript. In addition to allowing us to maintain objectivity, it also allows for the small possibility that triggering status epilepticus with flurothyl 13 days earlier may have led to some lingering ictal activity.

Multiple studies have demonstrated the dynamic nature of theta oscillations during epochs of movement in rodents (24, 88, 89). One additional limitation of our study is that we did not capture specific velocity or acceleration of the rats while recording oscillatory activity. Instead, analyses between groups were made in epochs that included similarly behaving and moving rats. We hope to include tracking software in future studies to address any issues related to movement speed.

One interesting unanswered question remains how stimulation improved outcome. We had initially hypothesized that 2 weeks of stimulation would lead to lasting improvements in neural activity as measured by changes in oscillations. However, we did not observe a long-term benefit of stimulation on theta or broadband power. The effects could not be related directly to the stimulation as in our previous studies (29, 33), as there was a 1-month gap between stimulation and behavior. Perhaps interesting to consider is that the efficacy of both RNS and DBS in epilepsy patients improves with time, even without changes to the stimulation paradigm (13, 14). These clinical data, along with our current data, suggest that repeated stimulation may lead to changes in neuroanatomy or connectivity that may explain the link between stimulation and the clear evidence of plasticity. These neuroanatomical changes could include a reduction in cell death, a reduction in neuroinflammation, or even change in neurotransmission. As tissue from these current experiments has significant artifact due to the number of implanted electrodes and, in many cases damage to the tissue caused by the removal of the implant, it is not suitable for a rigorous analysis of anatomical changes. Future studies should consider including animals implanted only with stimulating electrodes with the express goal of evaluating lasting histological changes that may inform the mechanism of action of septal DBS as it pertains to the long-term benefits of stimulation. In addition, a longitudinal genomics analysis to look at expression of genes related to neuroplasticity and neurotransmission before, during and after periods of neuromodulation could be highly informative.

Finally, animals were stimulated predominantly during the epileptogenic period. Our premise was that the induction of status epilepticus with pilocarpine served as the initial seizure events. However, one might argue that patients would not receive anti-seizure medication until they were diagnosed with recurring spontaneous seizures. Invasive neuromodulation would not be an option until a patient has been deemed refractory. However, the current data serve as a proof-of-concept that early intervention can lead to lasting changes in disease progression and outcome as animals benefitted from neuromodulation. Therefore, to bridge the translational gap, future studies should effects of stimulation occurring at least several weeks following the detection of spontaneous seizures and then to compare whether stimulation in these rats alters the progression of the disease similar to our observations in the current data set. Results from this type of study would enhance translatability to the clinical setting where patients have already experienced several years of seizures, prior to being classified as refractory to treatment.

## Conclusion

We now demonstrate in a rodent model of TLE that the MSN and theta rhythm represent a neuromodulation target that have the potential to both reduce markers of epilepsy while also improving cognitive outcome. These data are especially exciting, as neither rats receiving Fixed or Burst stimulation performed differently from Vehicle, whereas untreated Pilo rats had significant increases in spikes, a reduction in seizure threshold and demonstrated impaired performance on both the NOR and Barnes maze tasks. Furthermore, these data provide further support toward growing evidence that early intervention in patients with TLE results in superior outcomes. In the clinic, many patients do not meet criteria for resection, and while RNS and DBS of the ANT can lead to significantly improved seizure control over time, these therapies are not designed to address cognitive comorbidities and are often utilized decades after diagnosis. Our results represent a significant step toward identifying a promising DBS therapy for refractory patients with cognitive impairments, while also supporting the potential benefits of earlier intervention.

## Data Availability Statement

The raw data supporting the conclusions of this article will be made available by the authors, without undue reservation.

## Ethics Statement

The animal study was reviewed and approved by Office of Research Animal Care Program (IACUC).

## Author Contributions

AI contributed to all facets of the experiment including experimental design and data collection and analysis as well as writing. AS, KO, and GD each were involved with data collection and analysis as well as manuscript editing. SC, AE, and KS were involved with experimental design, data analysis, and editing. GG was involved in all aspects of the study including design, data collection and analysis, and writing. All authors contributed to the article and approved the submitted version.

## Funding

This study was supported by the National Institute of Health (NINDS NS084026) and the Bronte Epilepsy Foundation.

## Conflict of Interest

The authors declare that the research was conducted in the absence of any commercial or financial relationships that could be construed as a potential conflict of interest.

## Publisher's Note

All claims expressed in this article are solely those of the authors and do not necessarily represent those of their affiliated organizations, or those of the publisher, the editors and the reviewers. Any product that may be evaluated in this article, or claim that may be made by its manufacturer, is not guaranteed or endorsed by the publisher.
